# Management of a growing Skeletal Class II Patient: A Case Report

**DOI:** 10.5005/jp-journals-10005-1187

**Published:** 2013-04-26

**Authors:** Narendra Shriram Sharma

**Affiliations:** Assistant Professor, Department of Orthodontia, SP Dental College and Hospital, Wardha, Maharashtra, India

**Keywords:** Growing skeletal class II, ‘Two phase’ management, Pubertal growth spurt, Orthodontics and dentofacial orthopedics

## Abstract

Sagittal and transverse discrepancies often coexist in skeletal class II malocclusions. Orthopedic growth modification can work well in such cases, provided that the remaining pubertal growth is adequate and that the clinician can provide timely treatment to coincide with the peak growth period.

The transverse discrepancy is generally corrected first, establishing a proper base for the sagittal correction to follow. For example, in a skeletal class II case with a narrow maxillary arch and retrusive mandible, maxillary expansion is performed initially to facilitate functional mandibular advancement. The present article illustrates an exception to this rule, in a case where sagittal correction was undertaken before transverse correction to make optimal use of the patient's pubertal growth spurt in first phase followed by a second phase of fixed appliance therapy during adolescence to achieve optimal results.

**How to cite this article:** Sharma NS. Management of a growing Skeletal Class II Patient: A Case Report. Int J Clin Pediatr Dent 2013;6(1):48-54.

## INTRODUCTION

Whatever, the type of appliance that is used or the kind of growth effect that is desired; if growth is to be modified, the patient has to be growing. Growth modification must be done before the adolescent growth spurt ends. In theory, it could be done at any point up to that time. The ideal timing remains somewhat controversial but the recent research has clarified the indications for treatment at various ages. Unfortunately, although most anterior-posterior and vertical jaw discrepancies can be corrected during the primary dentition years, relapse occurs because of continued growth in the original disproportionate pattern. If children are treated very early; they usually need further treatment during the mixed dentition and again in the early permanent dentition to maintain the correction. For all practical purposes, early orthodontic treatment for skeletal problems is mixed dentition treatment; and a second phase of treatment during adolescence will be required.^1^ Sagittal and transverse discrepancies often coexist in skeletal class II malocclusions.^2-4^ Orthopedic growth modification can work well in such cases, provided that the remaining pubertal growth is adequate and that the clinician can provide timely treatment to coincide with the peak growth period.^5,6^

The transverse discrepancy is generally corrected first, establishing a proper base for the sagittal correction to follow.^7,8^ For example, in a skeletal class II case with a narrow maxillary arch and retrusive mandible, maxillary expansion is performed initially to facilitate functional mandibular advancement.^7,9^ The present article illustrates an exception to this rule, in a case where sagittal correction was undertaken before transverse correction to make optimal use of the patient's pubertal growth spurt in first phase followed by a second phase of fixed appliance therapy

## DIAGNOSIS AND ETIOLOGY

**Fig. 1:**
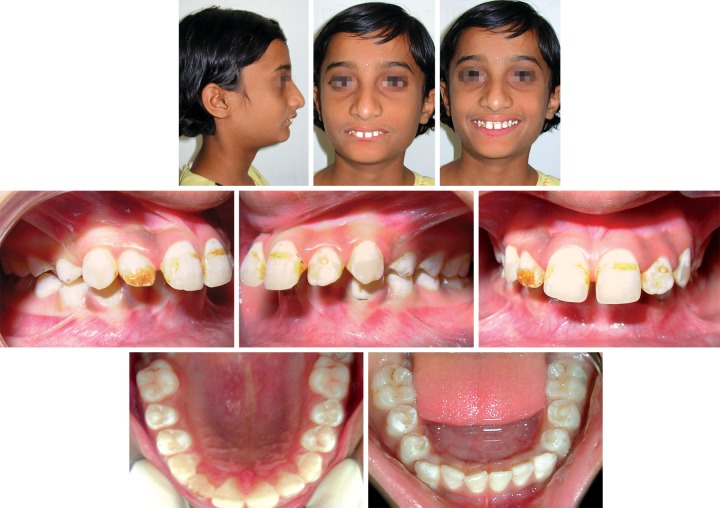
Pretreatment extraoral and intraoral photographs

**Fig. 2:**
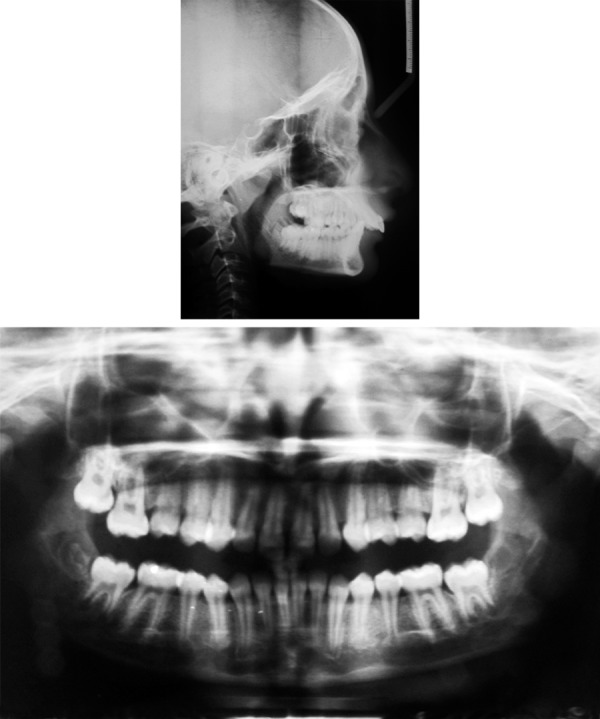
Pretreatment cephalogram and OPG

**Fig. 3:**
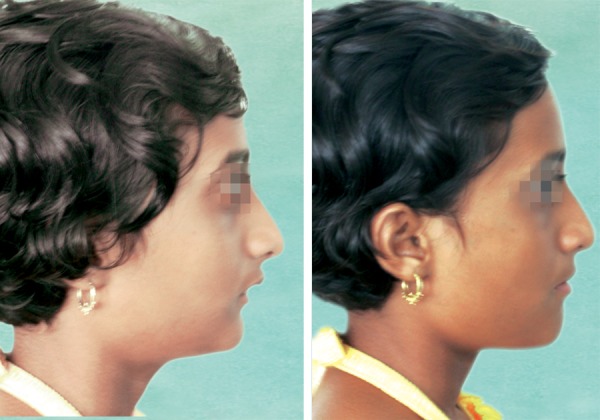
A positive VTO

A 12-year-old female presented with the chief complaint of protrusive upper front teeth. She exhibited a convex profile, an acute nasolabial angle, a protrusive upper lip, a trapped lower lip and a deficient chin (Fig. 1). The incompetent lips, reduced mandibular plane, and excessive incisal exposure and decreased lower anterior facial height all indicated a horizontal growth pattern. All permanent teeth were present except for the unerupted third molars. The canine and incisor relationships were class II. The maxillary anterior teeth were severely proclined, and the overbite was excessive (10 and 5 mm respectively). Midlines were coincident (Fig. 1). Cephalometric analysis confirmed the diagnosis of a division 1 malocclusion on a skeletal class II base, with a horizontal growth pattern and a marked mandibular retrusion (Table 1). Evaluation of the patient's cervical radiographs indicated that she was at the peak of the pubertal growth spurt, with considerable growth remaining (Fig. 2). In addition to this patient showing positive clinical VTO (Fig. 3).

## TREATMENT PLAN

Since she was in the pubertal growth spurt, limited treatment objectives, consisting of improving the skeletal jaw relationship as much as possible by growth modification and correcting the occlusal discrepancies by dentoalveolar compensation, were considered. The pubertal growth status of a patient is more critical for sagittal correction and because the patient was at the peak of pubertal growth, we decided to carry out the sagittal and the transverse correction simultaneously with a functional orthopedic approach by adding an expansion screw. A fixed twin block appliance was chosen to stimulate the forward mandibular growth.^10,11^

**Table 1:** **Table 1:** Pretreat cephalometric analysis

	*Mean*	*Pretreatment*
	*Maxilla to cranium*	
SNA angle	82 ± 2°	78°
N perp. Pt. A (mm)	0 ± 2 mm	–5 mm
Eff. max length		90 mm
	*Mandible to cranium*	
SNB angle	80 ± 2°	72°
N perp. – Pog (mm)	0 mm	–12 mm
Eff. mand. length (mm)		105 mm
N Pog – FH angle	87.8°	82 mm
	*Maxilla to mandible (skeletal)*	
ANB angle	2 ± 2°	6°
Wits (mm)	0 mm	4 mm
	*Vertical relationship*	
Y-axis angle	53-66°	55°
Facial axis angle	87.8°	82°
FMA angle	25°	18°
GoGn – SN	32°	22°
Occlusal to SN angle	9.3°	10°
UFH/LFH		72 mm/48 mm
	*Maxillary dental*	
UI to NA (angle)	22°	35°
UI to NA (mm)	4 mm	8 mm
UI to Pt. a vertical (mm)	2.7 mm	10 mm

This was to be followed by fixed-appliance therapy for simultaneous intrusion and retraction of the anterior teeth and finishing and detailing of the occlusion. The specific treatment objectives were to (1) correct the skeletal AP discrepancies with improvement of the soft-tissue profile (2) establish positive overjet and overbite, (3) establish class I molar and canine relationships; (4) eliminate the maxillary and mandibular arch length discrepancies and (5) follow-up the remaining growth to assess the need for further treatment. These changes were expected to greatly improve her facial profile and ensure the long-term stability of the treatment results.

**Fig. 4:**
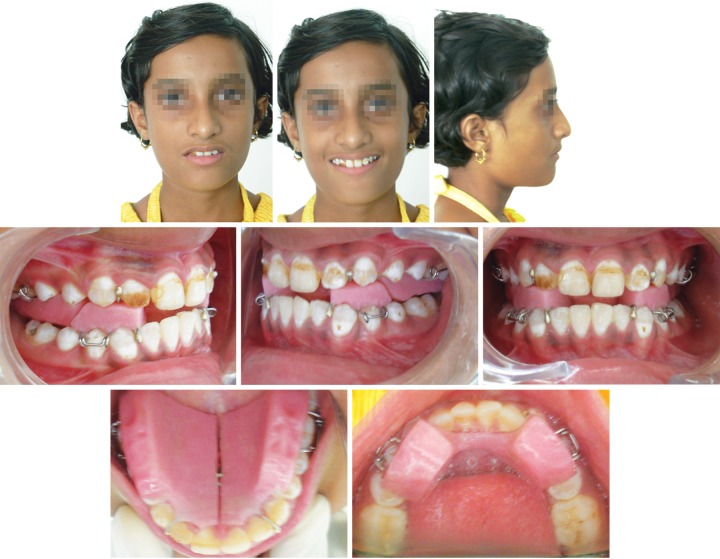
Twin block appliance in place

## TREATMENT PROGRESS

Treatment began with a bite for the twin block appliance with a 7 mm sagittal advancement and a 5 mm vertical opening in the premolar region (Fig. 4). The twin block appliance was fabricated with a maxillary expander placed on the maxillary arch. The patient was instructed to wear the appliance full-time except during meals and contact sports. After 6 months of wear, the pterygoid response was achieved and trimming was started.

After 11 months of good compliance, the patient showed a class I molar relationship with no dual bite and a considerably improved facial profile. After removal of the appliance, we noted a class I molar relationship, an overjet of 2 mm, and increases of 2 and 1.5 mm in the maxillary intercanine and intermolar widths respectively. The increased arch width in the canine regions had removed the occlusal interferences and settled the canines into a class I relationship with adequate buccal clearance. She practiced upper-lip exercises and an active anterior lip seal throughout the orthopedic treatment period.

**Fig. 5:**
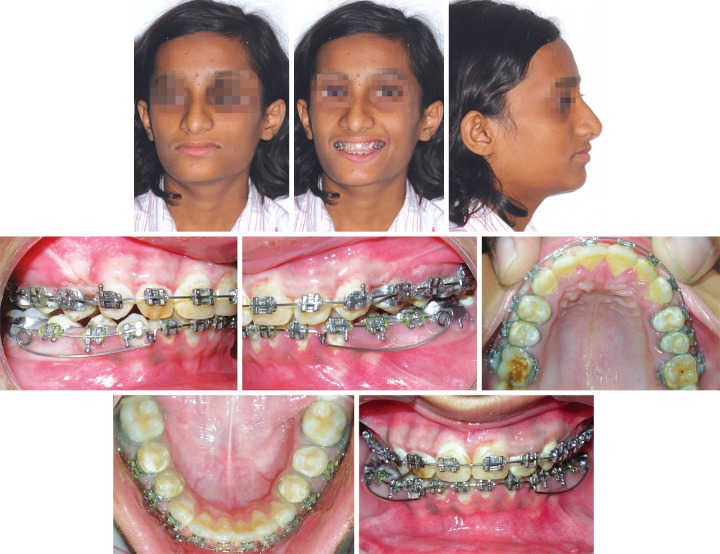
Preadjusted appliance in place

MBT-prescription 0.018" brackets were then bonded. For the first 7 months of fixed-appliance therapy, we used a removable transpalatal arch to maintain the vertical anchorage and sagittal expansion at the maxillary first molars, as well as 4.5 oz class II elastics to retain the sagittal correction. A utility arch was placed to intrude and retract the maxillary anterior teeth, closing the spaces. A Marcotte 3-piece intrusion arch was placed to intrude and retract the mandibular anterior teeth, closing the spaces (Fig. 5). After 10 months of fixed appliance treatment, the patient was highly satisfied with the treatment results.

**Fig. 6:**
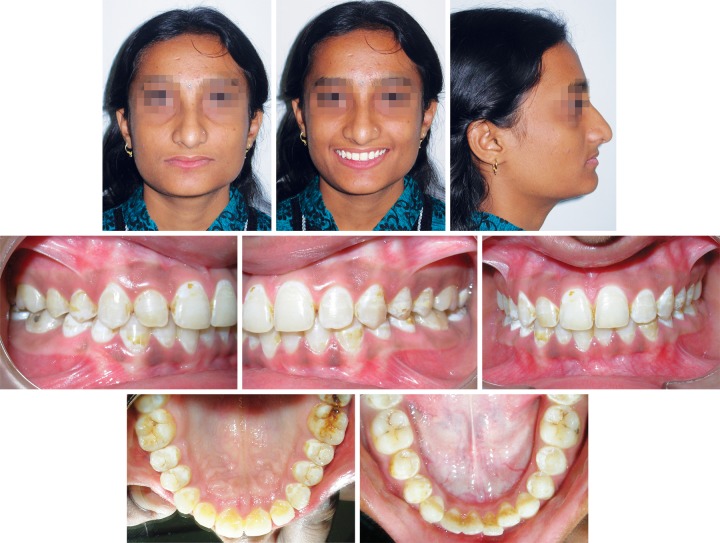
After debonding, preadjusted appliance

The fixed appliances were debonded for a total treatment time of 14 months (Fig. 6). The total treatment time was approximately 25 months. The retainers were the thermoplastic type and used full-time, except during meals and brushing, for the first 12 months. After this period, the retainers were switched to nocturnal use only for another 12 months.

## TREATMENT RESULTS

All treatment objectives were achieved. The anterior lip trap was corrected, and satisfactory dental alignment, normal overjet and overbite, and ideal class I molar and canine relationships on both sides were established. The overall facial balance was greatly improved. The post-treatment extraoral photographs showed a relaxed lip closure and an esthetically pleasing smile with a favorable smile arc. The patient was satisfied with her teeth and profile.

**Fig. 7:**
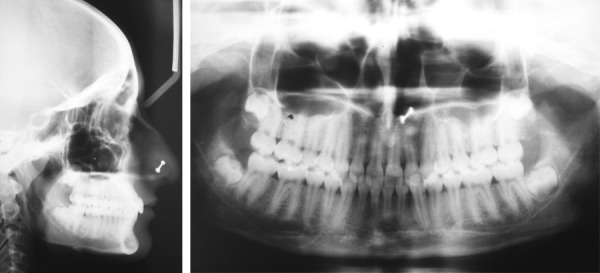
Post-treatment cephalogram and OPG

In the panoramic radiograph (Fig. 7), root parallelism was good, and no apical resorption was observed. The mandibular third molars were well-developed and positioned. The cephalometric analysis (Table 2) indicated that the AP relationship of the basal bone was improved.

**Fig. 8:**
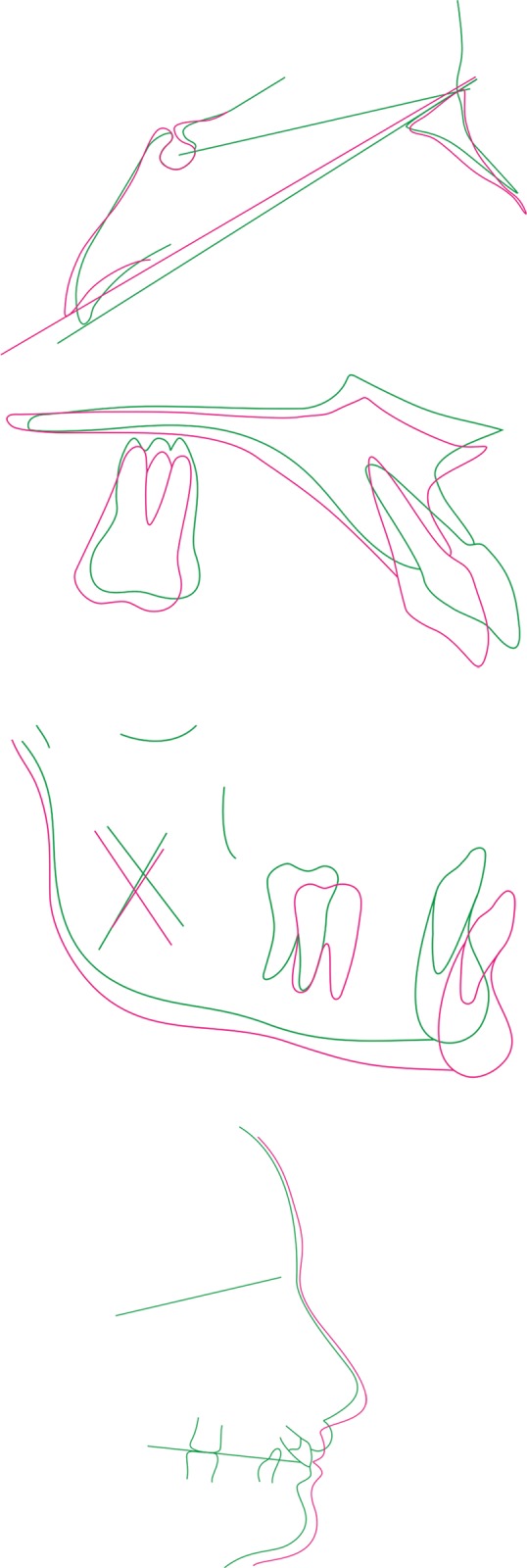
Superimposition of maxilla and mandible

Superimposition of the cephalometric tracings revealed a restriction in maxillary growth and considerable forward movement of the chin, resulting in a harmonious basal relationship (Fig. 8). Other factors contributing to the correction included sagittal and vertical maintenance of the maxillary molars, intrusion and retraction of the maxillary anterior teeth and counterclockwise rotation of the occlusal plane.

**Table 2:** **Table 2**: Post-treatment cephalometic analysis

	*Mean*	*Post-treatment*
	*Maxilla to cranium*	
SNA angle	82 ± 2°	78°
N perp. Pt. A (mm)	0 ± 2 mm	–5 mm
Eff. max length		90 mm
	*Mandible to cranium*	
SNB angle		75°
N perp. – Pog (mm)	0 mm	–7 mm
Eff. mand. length (mm)		1105 mm
	*Maxilla to mandible (skeletal)*	
ANB angle	2 ± 2°	3°
Wits (mm)	0 mm	0 mm
	*Vertical relationship*	
Y-axis angle	53-66°	60°
Facial axis angle	87.8°	87°
FMA angle	25°	21°
GoGn – SN	32°	26°
Occlusal to SN angle	9.3°	10.5°
UFH/LFH		73 mm/52 mm
	*Maxillary dental*	
UI to NA (angle)	22°	22°
UI to NA (mm)	4 mm	5 mm
UI to Pt. a vertical (mm)	2.7 mm	5.5 mm
UI to SN (angle)	102 ± 2°	103°

## DISCUSSION

Class II malocclusions might have any number of combinations of skeletal and dental components. So, identifying and understanding the etiology and expression of a class II malocclusion and forming the correct differential diagnosis are essential for its correction, whether it is orthodontic, orthopedic, surgical or a combination of these modalities. From an etiologic perspective, few malocclusions have one specific cause; more often, they are the result of a combination of many factors in the inherent predetermined growth potential of each patient. Thus, for any malocclusion, especially a skeletal malocclusion, multiple-factor treatment is superior to that of a single factor. Previous studies regarding morphologic characteristics of skeletal class II malocclusion present various and contradicting opinions. But it is generally believed that a skeletal class II malocclusion is often caused by some combination of mandibular deficiency and maxillary excess. The success of combination therapy (distal jet and Jasper jumpers) in class II malocclusion suggests that the problem is not concentrated in a single jaw (maxilla or mandible), so a bimaxillary treatment design might achieve a better result. Here, we treated a developing skeletal class II patient with combination therapy using a twin block and SWA.

In this patient, the satisfactory occlusal and esthetic results were due to significant dentoalveolar compensation and excellent patient compliance with the twin block. The changes contributing most to the correction of the initial dental and skeletal AP discrepancy were forward mandibular growth, maxillary incisor retroclination and distal *en masse* movement of the maxillary dentition with concurrent alveolar remodeling. These changes produced a counterclockwise rotation of the occlusal plane as expected and improved the soft-tissue profile, with retrusion of the upper lip and slight protrusion of the lower lip. Although the mandible rotated slightly clockwise (1.0°) still resulted in a class I occlusion. Although the upper anterior intrusion and increased tonicity of the upper lip reduced the incisal exposure, a complete passive lip seal could not be achieved. On retrospective analysis, however, the treatment plan was justified by the results achieved.

## CONCLUSION

A good esthetic and functional result was achieved for this patient. This was achieved by employing a stepwise functional advancement and two phase treatment protocol that was tailored specifically to this patient's needs. During the treatment, oral hygiene was continually reinforced and treatment mechanics adjusted to simplify oral hygiene.

This approach took advantage of the patient's pubertal growth spurt to achieve a sagittal correction that otherwise would have been a missed opportunity. Our case exemplifies the need for individualized treatment planning rather than a cook-book approach in the management of dentofacial
